# The multiplicity problem in linkage analysis of gene expression data – the power of differentiating *cis*- and *trans*-acting regulators

**DOI:** 10.1186/1753-6561-1-s1-s142

**Published:** 2007-12-18

**Authors:** Baisong Huang, Jagadish Rangrej, Andrew D Paterson, Lei Sun

**Affiliations:** 1 Research Institute, Hospital for Sick Children, 555 University Avenue, Toronto, Ontario, M5G 1X8, Canada; 2 Department of Public Health Sciences, University of Toronto, 155 College Street, Toronto, Ontario, M5T 3M7, Canada; 3 Department of Statistics, University of Toronto, 100 St. George Street, Toronto, Ontario, M53 3G3, Canada

## Abstract

In this report, we focused on the multiplicity issue in Problem 1 of Genetic Analysis Workshop 15. We investigated and compared the performance of the stratified false-discovery rate control method with the traditional aggregated approach, in an application to genome-wide linkage analyses of single-nucleotide polymorphism-to-gene expression data. We showed the importance of utilizing the available map information and demonstrated the power gained by conducting false-discovery rate control separately for *cis *and *trans *regulators under three different frameworks: fixed rejection region, fixed false-discovery rate, and fixed number of rejections.

## Background

The routine use of multiple-hypothesis testing in inference for large-scale genetic and genomic data has generated controversies and discussions about appropriate ways to adjust for the multiplicities. The conventional control of family-wise error rate (FWER) strictly regulates the probability of type I error but with a considerable loss of power. The recent methodology based on false-discovery rate (FDR) control [1] is an alternative that provides better power yet controls the occurrence of false positives, and its use has become common in the analyses of microarray gene expression data.

Of particular interest here is the use of auxiliary or prior information in the FDR setting. The stratified FDR method [2] was chosen for this analysis because the prior information required is rather general and does not need any distribution assumptions. Sun et al. [2] investigated the performance of the method under two frameworks and showed that under the fixed rejection region framework, the aggregated FDR is a weighted average of the stratum-specific FDRs; under the fixed FDR framework, the stratified approach identifies more true positives. Recently, Greenwood et al. [3] considered the stratification principle under a third framework: fixed number of rejections. They demonstrated that the stratified approach provides an FDR control at a lower rate.

In this report, we focused on the stratified FDR method [2] and applied it to Problem 1: Genetics of Gene Expression in Humans in Genetic Analysis Workshop 15. We considered the two-stage design of Morley et al. [4] in which genome-wide linkage analyses of single-nucleotide polymorphism (SNP)-to-gene expression data were first carried out and significant results were then selected for further studies. We were particularly interested in the selection procedure and comparisons of the performance between the stratified FDR method and the traditional aggregated FDR control. The prior information considered here is the map distance between a SNP and a gene whose expression is the trait.

## Methods

We first performed expression quantitative trait linkage (eQTL) analysis using MERLIN regress v 1.0.1 [5,6]. We zeroed out the genotypes of the child as well as those of the grandparents when a Mendelian inconsistency was detected. All other genotypes were retained. Marker allele frequencies were estimated from the data and single-point linkage analyses of gene expression data were performed for all pairs of 3554 genes and 2819 SNPs, excluding the ones (genes or SNPs or both) on the sex chromosomes. We then stratified the linkage results by the map distance between each SNP and the gene. Gene positions were obtained from Build 36.1 of the UCSC Genome Browser [7]. SNP locations were obtained from Build 126 of dbSNP [8] (on Build 36.1 of the human genome). We used the definition of Morley et al. [4] in which *cis *regulators are the SNPs within 5 Mb of genes and *trans *regulators are the remaining SNPs. Stratum 1 contained *cis *SNPs and stratum 2 contained *trans *SNPs, and the aggregated group included all SNPs ignoring stratification. We used *m *to denote the total number of hypotheses among which *m*_0 _were true nulls, *R *to denote the number of rejections, and *p*_(*i*)_, *i *= 1,...,*m *to denote the linkage p-values while *p*_(1) _≤ *p*_(2) _≤ ... ≤ *p*_(*m*) _were the ordered *p*-values. We used superscript (*k*), *k *= 1, 2 as the stratum indicator. Finally, we applied the stratification principle [2] under the following three frameworks.

### Framework I: fixed rejection region

This framework chooses the rejection region in advance, i.e., it rejects all hypotheses with unadjusted *p*-values less than a pre-determined *α *value, e.g., *α *= 0.01%. The corresponding FDR level among the *R *positives can then be estimated by $F\hat{D}R\left(\alpha \right)=\left(m{\hat{\pi }}_{0}\alpha \right)/R$, where *R *= {*p*_(*i*) _≤ *α*}, ${\hat{\pi }}_{0}$ is an estimate of the proportion of null hypotheses, *π*_0 _= *m*_0_/*m*, e.g., ${\hat{\pi }}_{0}$(*λ*) = #{*p*_*i *_> *λ*}/(*m*(1 - *λ*)) with *λ *= 0.5. The rejection procedure remains the same for the stratified method using the same *α *level (*R*^(1) ^+ *R*^(2) ^= *R*). However, the estimates of FDR among *R*^(1) ^and *R*^(2) ^can be considerably different from the aggregated FDR, with one stratum estimate closer to 0 and the other closer to 1. For both cases, one thus obtains more information on the specificity of the results.

### Framework II: fixed FDR

Under this framework, the targeted FDR level is pre-chosen at a *γ *level, e.g., *γ *= 5%. Storey [9] showed that controlling FDR at the *γ *level is equivalent to rejecting all tests with *q*-values ≤ *γ*, and the *q*-values can be estimated by ${\hat{q}}_{\left(i\right)}=\min\{{\hat{\pi }}_{0}mp_{\left(i\right)}/i,{\hat{q}}_{\left(i+1\right)}\}$, and ${\hat{q}}_{\left(m\right)}={\hat{\pi }}_{0}p_{\left(m\right)}$. This method is equivalent to the FDR adjusted *p*-value method [10,11]. To fairly compare the performance of the stratified FDR method with the aggregated one, we controlled the FDR at the same level for both Strata 1 and 2 and the aggregated group using the above *q*-value method. The objective was to show that the total number of rejections *R*^(1) ^+ *R*^(2) ^under stratification is greater than *R *under aggregation.

### Framework III: fixed number of rejections

In this case, the total number of significant results *R *that merits further study is pre-determined based on, for example, the budget and capacity of a particular chip platform. Without stratification, the choice of *R *hypotheses is straightforward, i.e., the *R *tests with the smallest *p*-values: *p*_(1)_,...,*p*_(*R*)_. The corresponding FDR level can be estimated by $F\hat{D}R=\left(m{\hat{\pi }}_{0}p_{\left(R\right)}\right)/R$. Under stratification, one needs to find the optimal configuration of *R*^(1) ^and *R*^(2) ^such that *R*^(1) ^+ *R*^(2) ^= *R *and the overall FDR is minimized, where $F\hat{D}R_{stra}=\left(m^{\left(1\right)}{\hat{\pi }}_{0}^{\left(1\right)}p_{\left(R^{\left(1\right)}\right)}^{\left(1\right)}+m^{\left(2\right)}{\hat{\pi }}_{0}^{\left(2\right)}p_{\left(R^{\left(2\right)}\right)}^{\left(2\right)}\right)/R$. The goal is to show that stratification leads to a smaller FDR rate given the same number of positives allowed. More importantly, the configuration of *R*^(1) ^and *R*^(2) ^obtained using stratification can differ markedly from the aggregation case. Without stratification, the distribution of *R *rejections between the two strata is roughly proportional to the number of hypotheses in each stratum; while with stratification, the stratum with smaller *π*_0 _(less noise) and higher power to detect true signals proportionally rejects more hypotheses.

## Results

Among the 9,069,390 tests with valid results, more than half (5,000,428) had *p*-values equal to 1. We eliminated the tests with *H*^2 ^= 0, where *H*^2 ^is the estimated locus-specific heritability, because we observed a significant association between *p*-value = 1 and *H*^2 ^= 0 (*p *< 0.0001). Among the remaining 3,855,428 tests, 16,018 were in the *cis *stratum and 3,839,410 in the *trans *stratum.

The distributions of the aggregated *p*-values and partitioned *p*-values in *cis *and *trans *strata were roughly uniform. (Figures illustrating these distributions are available at .) As expected, the *cis *stratum contained a relatively higher proportion of true signals than the *trans *stratum, which was further confirmed by the smaller estimates of *π*_0 _shown in Table 1 (88.18% vs. 95.06%) and the higher density of small *p*-values close to zero. Results in Table 1 clearly demonstrated that the stratified method outperformed the aggregated approach under all three frameworks. For example, under the fixed rejection region approach, among the 8043 rejections with aggregated FDR of 4.56%, 129 belonged to the *cis *stratum with a much lower stratum-specific FDR of 1.09%. In fact, among the expected 366 false positives, only 1 was expected to be from the *cis *group, a clear gain of information by the use of stratification. Under the fixed FDR framework, the stratified method provided 57 more true positives while controlling the FDR at the same rate as the aggregated approach. Under the fixed number of rejections framework, e.g., *R *= 2000 in Table 1, the aggregated FDR method rejected the tests with the smallest *p*-values regardless of the map information. Among the 2000 rejections, 55 belonged to *cis *stratum and 1945 to *trans *stratum corresponding to an overall $F\hat{D}R$ = 2.05%. In contrast, the stratified FDR approach allowed different rejection configurations in the two strata, with *R*^(1) ^= 133 and *R*^(2) ^= 1867 being the optimal one corresponding to an overall $F\hat{D}R$ = 2.00%. Besides the obvious advantage of achieving a smaller FDR, the stratified method allocated proportionally more rejections out of the 2000 to the *cis *stratum (133 vs. 55), a preferable result given the different characteristics of the *cis *and *trans *regulators. Other choices of the total number of rejections, i.e., *R *= 500, *R *= 1000 and *R *= 8000 gave similar results. (Figures illustrating these results are available at .)

**Table 1:** Summary statistics and results under the three frameworks

		Stratum	
		
Parameter	Aggregated	*cis*	*trans*
Summary statistics			
No. tests	3,855,428	16,018	3,839,410
${\hat{\pi }}_{0}$ (%)	95.03	88.18	95.06
Minimal *q*-value	2.23 × 10^-7^	1.12 × 10^-9^	4.44 × 10^-7^
Framework I, *α *= 0.0001			
No. rejections	8,043	129	7,914
$F\hat{D}R$ (%)	4.56	1.09	4.61
E [No. false positives]	366	1	365
Framework II, *γ *= 0.05			
No. rejections	8,541	339	8,262
E [No. true positives]	8,114	322	7,849
Framework III, *R *= 2,000			
Rejection without stratification	2000	55	1945
Rejection with stratification	2000	133	1867
			
Overall $F\hat{D}R$ (%)	2.05	2.00	

## Discussion

The stratified FDR method of Sun et al. [2] provides a simple way of incorporating the available auxiliary or prior information to improve power in the context of multiple hypothesis testing. We applied their stratification principle to the linkage analyses of gene expression data of Problem 1 under three different frameworks. Framework II represents the traditional view of type I error control in which a desirable error rate is pre-determined. However, the nominal level could be too optimistic for a given data set, leading to no rejections, or too liberal, resulting in too many significant results for follow up studies. Therefore, in many applications Frameworks I and III are more applicable and meaningful. Results of our analyses demonstrated clearly that it is advantageous to utilize the available map information under any of the three frameworks considered. However, the stratified method did not outperform the aggregated approach by as large a margin as we had expected given the known characteristic difference between *cis *and *trans *regulators. One possible explanation is that the large number of *trans *regulators overwhelms the potential gain using the current measure of efficiency.

To demonstrate that the smaller FDR estimate in the *cis *stratum was not an artifact of sampling variation or the highly skewed subset size of the tests but the true biological difference in *cis*- and *trans*-acting loci, we randomly sampled a set of 16,018 *p*-values as the "*cis*" stratum and performed the corresponding Framework I analyses, and this procedure was repeated 20 times independently. The means of the estimated *π*_0 _and FDR were 94.76% (SE = 0.67%) and 4.73% (SE = 0.9%), respectively. Accounting for the sampling variation, these values differed significantly from those obtained using the available map information (${\hat{\pi }}_{0}$ = 88.18%, $F\hat{D}R$ = 1.09%), while they were essentially the same as those under aggregation (${\hat{\pi }}_{0}$ = 95.03%, $F\hat{D}R$ = 4.56%). In addition, the average number of rejections (i.e., tests with *p*-values < 0.0001) over the 20 random samples is 33.25 (SE = 6.6), which is close to the expected number of rejections (8043*16018/3855428 = 33.42) in a random sample of 16018 *p*-values; while the original *cis *stratum, defined based on the map distance, had 129 rejections (i.e., higher density of small *p*-values than a random sample).

In our analyses, we used the criterion of Morley et al. [4] to define the *cis *and *trans *regulators. Other definitions are possible and will change the FDR results quantitatively but not qualitatively. This is clearly demonstrated by the results shown in Table 2 for which we redefined *cis *regulators as the SNPs within 3, 10, or 20 Mb of genes. The more interesting and challenging question is the identification of the optimal stratum indicator. Searching through a list of candidates is an obvious but biased approach because it added another level of multiplicity [2].

**Table 2:** Results of using different distance criterion to define *cis *regulators

	3 Mb		10 Mb		20 Mb	
			
Parameter	*cis *(<)	*trans *(≥)	*cis *(<)	*trans *(≥)	*cis *(<)	*trans *(≥)
No. tests	9,699	3,845,729	29,827	3,825,601	52,853	3,802,575
${\hat{\pi }}_{0}$ (%)	87.68	95.05	89.32	95.08	89.62	95.11
Framework I, *α *= 0.0001						
No. rejections	69	7,974	204	7,839	302	7,741
$F\hat{D}R$ (%)	1.23	4.58	1.31	4.64	1.57	4.67
E [No. false positives]	1	365	3	363	5	361
Framework II, *γ *= 0.05						
No. rejections	200	8,398	519	8,113	761	7,927
E [No. true positives]	190	7,978	493	7,707	723	7,531
Framework III, *R *= 2,000						
Rejection without stratification	29	1971	90	1,910	127	1,873
Rejection with stratification	67	1933	184	1,816	222	1,778
						
Overall $F\hat{D}R$ (%)	2.03		1.99		1.99	

It is also possible to exploit the available map information in other ways. For example, one could apply the weighted *p*-value method [12] by using the map distance as the weighting factor. That is, perform the FDR control on weighted *p*-value *p*_*w *_= *p/w*, where *p *is the original linkage *p*-value between a pair of SNP and gene, and *w *is the corresponding weight inversely proportional to the map distance. Similar to the stratification case above, the choice of a specific weighting scheme is not unique and the identification of the optimal one remains an open problem. In addition, the comparison and connection between the weighted *p*-value method and the stratified approach is of interest.

## Competing interests

The author(s) declare that they have no competing interests.
